# Prognostic Impact of Carvedilol vs. Metoprolol on Long-Term Outcomes in Patients with Heart Failure and Mildly Reduced Ejection Fraction

**DOI:** 10.3390/jcm15093347

**Published:** 2026-04-28

**Authors:** Kathrin Weidner, Michael Behnes, Marielen Reinhardt, Noah Abel, Alexander Schmitt, Felix Lau, Mohammad Abumayyaleh, Svetlana Hetjens, Henning Johann Steffen, Ibrahim Akin, Tobias Schupp

**Affiliations:** 1 Department of Cardiology, Angiology, Haemostaseology and Medical Intensive Care, Medical Faculty Mannheim, University Medical Centre Mannheim, 68167 Mannheim, Germany; 2 Department of Medical Statistics and Biomathematics, Medical Faculty Mannheim, University of Heidelberg, 68167 Mannheim, Germany

**Keywords:** heart failure with mildly reduced ejection fraction, HFmrEF, beta-blockers, pharmacotherapies, mortality

## Abstract

**Background:** Evidence regarding potential agent-specific differences among β-blockers in heart failure with mildly reduced ejection fraction (HFmrEF) remains limited. **Objective:** The present study sought to investigate the association of metoprolol versus carvedilol prescribed at hospital discharge with 30-month all-cause mortality and HF-related rehospitalization, and to explore potential effect modification by atrial fibrillation (AF). **Methods:** Consecutive patients hospitalized with HFmrEF between 2016 and 2022 were included. Exposure was β-blocker therapy at discharge (metoprolol succinate or carvedilol). Outcomes were analyzed using Kaplan–Meier estimates, multivariable Cox regression and propensity score matching. **Results:** Among 2109 patients discharged alive, 1625 (77.5%) received β-blockers (metoprolol *n* = 1033; carvedilol *n* = 283). Carvedilol recipients were younger (median 72 vs. 76 years) and more frequently had prior heart failure (44.2% vs. 33.2%). Thirty-month mortality occurred in 25.5% of metoprolol-treated and 31.8% of carvedilol-treated patients (unadjusted hazard ratio (HR) 0.77, 95% confidence interval (CI) 0.61–0.98; *p* = 0.031). This association was observed in patients without AF, but not in those with AF. After multivariable adjustments, the association remained directionally similar (adjusted HR 0.76, 95% CI 0.58–1.00). In the matched cohort (*n* = 246 per group), metoprolol was still associated with lower mortality (HR 0.65, 95% CI 0.46–0.93; *p* = 0.017). By contrast, HF-related rehospitalization did not differ significantly between the two groups. **Conclusions:** In this observational HFmrEF cohort, treatment with metoprolol at index hospital discharge was associated with lower 30-month mortality compared with carvedilol. Given the observational study design in line with the higher burden of comorbidities in patients discharged on carvedilol, further prospective studies are needed to clarify the impact of different β-blocker types in heart failure patients.

## 1. Introduction

Heart failure (HF) remains a leading cause of morbidity and mortality worldwide, with a growing burden on healthcare systems [1]. While the pathophysiology of HF with reduced ejection fraction (HFrEF) and preserved ejection fraction (HFpEF) is well-delineated, the subgroup of HF with mildly reduced ejection fraction (HFmrEF) has emerged as a clinical entity that warrants further exploration [2,3,4]. HFmrEF, characterized by a left ventricular ejection fraction (LVEF) between 41% and 49%, represents a heterogeneous group sharing features with both HFrEF and HFpEF patients [2,5]. Despite its increasing recognition, there remains a lack of robust clinical evidence on optimal management strategies for HFmrEF, leading to uncertainties in treatment decisions and guideline recommendations [2,6]. Several pharmacotherapies—including β-blockers, angiotensin converting enzyme inhibitors (ACEi), mineralocorticoid receptor antagonists (MRAs), and sodium–glucose co-transporter 2 inhibitors (SGLT2i)—were demonstrated to improve outcomes in HFrEF [2]. In HFmrEF, treatment effects appear heterogeneous; however, SGLT2 inhibitors have been shown to improve outcomes in patients with HFpEF and HFmrEF [7]. Whereas no randomized controlled trial (RCT) has yet demonstrated a prognostic benefit of β-blockers in HFmrEF, registry data suggest β-blockers may improve the outcomes of patients with HFmrEF [8]; however, the prognostic impact of β-blockers may depend on the type of β-blocker.

Metoprolol (β1-selective) and carvedilol (non-selective with additional α1-blockade) differ mechanistically, which may translate into distinct effects on haemodynamics, cardiac remodelling, and clinical outcomes in HFmrEF [9]. This question is clinically relevant; however, the only head-to-head RCT in HFrEF demonstrated agent-specific differences between carvedilol and metoprolol [9]. Moreover, an individual-patient data meta-analysis showed a β-blocker benefit in LVEF 40–49% without identifying a preferred agent [10], while contemporary reviews highlight the persistent evidence gap in HFmrEF.

Accordingly, this study aimed to evaluate the association between discharge treatment with metoprolol versus carvedilol and long-term outcomes in patients with HFmrEF.

## 2. Materials and Methods

### 2.1. Study Patients, Design and Data Collection

This retrospective single-centre cohort study was conducted at the University Medical Center Mannheim, Germany. All consecutive adult patients (≥18 years) hospitalized between 2016 and 2022 with documented HFmrEF during the index hospitalization were eligible for inclusion. HFmrEF was defined in accordance with the 2021 ESC Guidelines as a LVEF of 41–49% in the presence of signs and/or symptoms of HF. LVEF was assessed by transthoracic echocardiography during routine clinical care within the index hospitalization. Hospitalization was not restricted to acute decompensated HF as the primary diagnosis. Patients were included irrespective of the primary reason for admission, provided HFmrEF was documented during the index hospitalization.

The study was based on data from the “Heart Failure with Mildly Reduced Ejection Fraction Registry” (HARMER), a retrospective single-centre registry. The study was conducted in accordance with the Declaration of Helsinki and approved by the local ethics committee (approval number: 2022-818).

### 2.2. Inclusion and Exclusion Criteria

All consecutive patients aged ≥18 years with HFmrEF documented during the index hospitalization were eligible for inclusion. Inclusion required a LVEF of 41–49% assessed during the index stay. Patients who died during the index hospitalization were excluded, as exposure classification was based on β-blocker therapy at discharge. Furthermore, patients without β-blocker therapy at discharge and those receiving β-blockers other than metoprolol succinate or carvedilol were excluded from the final analysis.

### 2.3. Risk Stratification

For the present study, risk stratification was performed according to the type of β-blocker prescribed at hospital discharge. Documentation of β-blocker therapy was derived from the electronic hospital information system and discharge medication records. Patients were categorized based on treatment with either metoprolol succinate or carvedilol. Patients receiving β-blockers other than metoprolol succinate or carvedilol, as well as those without β-blocker therapy at discharge, were excluded from the present analysis. In addition, subgroup analyses were performed according to the presence or absence of atrial fibrillation (AF), as documented in the medical history or during index hospitalization.

### 2.4. Study Endpoints

The primary endpoint was 30-month all-cause mortality. The key secondary endpoint was rehospitalization for worsening HF. HF-related rehospitalization was defined as any readmission requiring intravenous diuretic therapy for worsening HF. This included admissions primarily due to HF exacerbation as well as admissions for other causes during which worsening HF was present at admission or developed during hospitalization. HF-related rehospitalizations were captured exclusively at our institution. Events occurring at external hospitals were not systematically recorded and may therefore have led to underestimation of rehospitalization rates.

### 2.5. Statistical Methods

Continuous variables were presented as medians with interquartile ranges (IQR). Categorical variables were given as absolute and relative frequencies. For baseline characteristics, standardized mean differences (SMD) reported as Cohen’s *d* were used to quantify the magnitude of differences between groups. Primary and secondary endpoints were compared using the Chi-square test or Fisher’s exact test. Kaplan–Meier survival analyses were performed to compare outcomes between treatment groups in the overall cohort and stratified by the presence or absence of AF. Univariable hazard ratios (HRs) with 95% confidence intervals (CIs) were calculated. Multivariable Cox regression models with forward selection were used to evaluate the association between β-blocker type and outcomes and adjusted for clinically relevant covariates. Patients with incomplete data were excluded from multivariable Cox regression analyses. The proportional hazards assumption was tested for all covariates using weighted Schoenfeld residuals to verify that the effect of each variable on the hazard remained constant over time. Schoenfeld residuals were derived at each event time as the difference between the observed covariate value of the individual experiencing the event and the expected value based on the corresponding risk set. A nonsignificant association between residuals and time was interpreted as evidence supporting the proportional hazards assumption, whereas a significant association indicated a potential violation.

To further address potential confounding by indication, propensity score matching was performed. A non-parsimonious multivariable logistic regression model was constructed including age, sex, body mass index, diabetes mellitus, chronic kidney disease, prior congestive HF, acute decompensated HF during index hospitalization, AF, coronary artery disease, New York Heart Association (NYHA) functional class, and relevant HF-directed pharmacotherapies at discharge. Patients were matched 1:1 using nearest-neighbour matching without replacement with a caliper width of 0.05. Within the matched cohort, Kaplan–Meier and univariable Cox regression analyses were performed. As a sensitivity analysis, inverse probability of treatment weighting (IPTW) based on propensity scores derived from a logistic regression model was applied. Stabilized weights were used and extreme propensity scores were trimmed to improve overlap between groups. Weighted Cox proportional hazards models were then used to investigate the association of metoprolol and carvedilol with regard to the primary endpoint.

All statistical tests were two-sided, and *p*-values ≤ 0.05 were considered statistically significant. Statistical analyses were performed using SPSS version 28 (IBM Corp., Armonk, NY, USA).

## 3. Result

### 3.1. Study Population

Between 2016 and 2022, 2228 patients were hospitalized with HFmrEF at our institution. After excluding 44 patients with incomplete follow-up and 75 patients who died during the index hospitalization, the final cohort comprised 2109 patients discharged alive. Of these, 1033 patients were discharged on metoprolol succinate and 283 on carvedilol (Table 1). Patients receiving carvedilol were younger (median 72 [IQR 60–81] vs. 76 [65–83] years, *d* = 0.823) and more frequently males (72.8% vs. 64.0%, *d* = 0.226). Prior congestive HF (44.2% vs. 33.2%, *d* = 0.256) and decompensated HF within 12 months (17.7% vs. 11.2%, *d* = 0.291) were more common in the carvedilol group, whereas chronic obstructive pulmonary disease was less prevalent (10.8% vs. 15.2%, *d* = 0.214). Rates of prior coronary artery disease (42.4% vs. 42.7%, *d* = 0.007) and prior myocardial infarction (25.8% vs. 25.8%, *d* = 0.002) were comparable. AF was present in 42.0% of carvedilol-treated patients and 47.0% of metoprolol-treated patients (*d* = 0.032). Ischemic cardiomyopathy was the leading HF etiology (59.7% vs. 64.9%, *d* = 0.021) in both groups. Median LVEF was 45% in both groups, whereas left ventricular end-diastolic diameter was slightly larger in patients receiving carvedilol (median 50 vs. 48 mm, *d* = 0.209). Moderate-to-severe tricuspid regurgitation was observed in 15–17% and mitral regurgitation in 11–14% of patients, without significant differences between the two groups. Coronary angiography was performed in approximately half of the cohort, with three-vessel disease present in 38.6% vs. 41.5%. Percutaneous coronary intervention during index hospitalization was more frequent in the metoprolol group (60.1% vs. 50.3%, *d* = 0.219). Baseline creatinine (1.09 vs. 1.07 mg/dL) and NT-proBNP levels (2370 vs. 2634 pg/mL) were similar between groups (Table 2).

At discharge, use of ACE inhibitors or angiotensin receptor blockers was comparable (58.3% vs. 56.0%), whereas MRAs (25.1% vs. 13.6%, *d* = 0.419), angiotensin receptor–neprilysin inhibitors (3.5% vs. 1.1%, *d* = 0.675), and SGLT2i (8.1% vs. 3.6%, *d* = 0.478) were more frequently prescribed for patients in the carvedilol group (Table 2).

### 3.2. Primary and Secondary Endpoints

**Figure 1:**
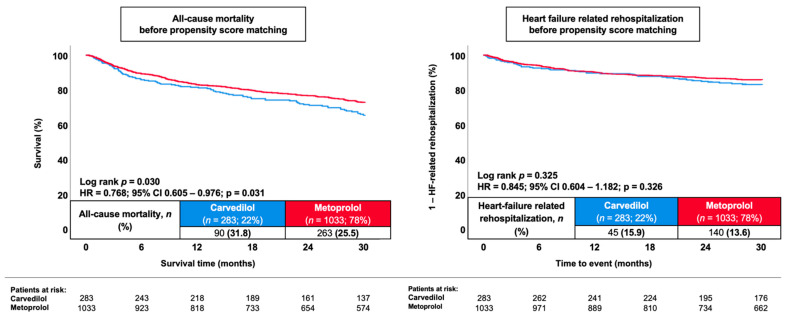
Kaplan–Meier curves comparing carvedilol and metoprolol succinate for 30-month all-cause mortality (**left panel**) and HF-related rehospitalization (**right panel**).

**Figure 2:**
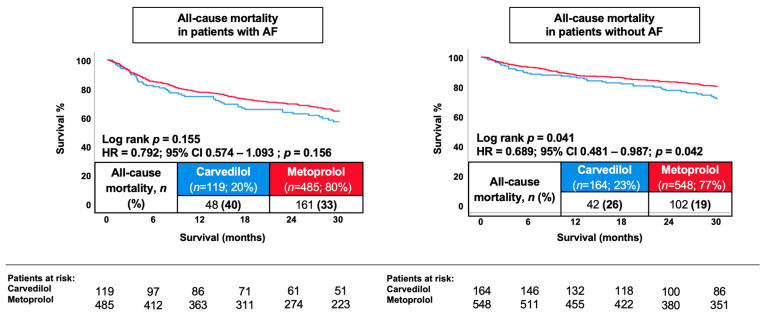
Kaplan–Meier curves illustrating the association between carvedilol and metoprolol succinate and 30-month all-cause mortality stratified by atrial fibrillation status (patients with AF (**left panel**); patients without AF (**right panel**)).

**Figure 3:**
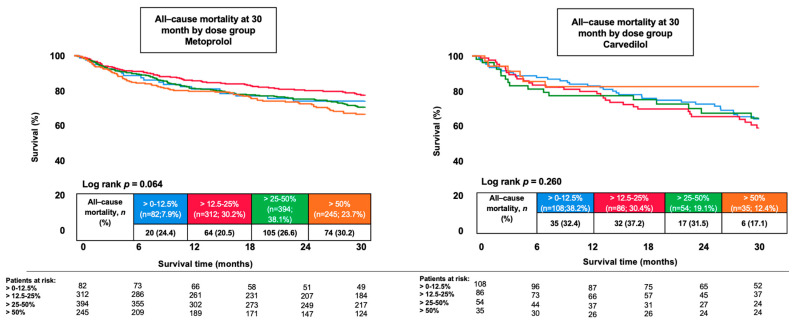
Kaplan–Meier curves illustrating 30–month survival according to percentage of target β-blocker dose prescribed at discharge.

During a median follow-up of 30 months, all-cause mortality was lower among patients discharged on metoprolol compared to carvedilol (25.5% vs. 31.8%; HR 0.77, 95% CI 0.61–0.98; *p* = 0.031; Figure 1). No statistically significant differences were observed for secondary outcomes. HF-related rehospitalization occurred in 13.6% of metoprolol-treated patients and 15.9% of carvedilol-treated patients (HR 0.85, 95% CI 0.60–1.18; *p* = 0.326). In subgroup analyses stratified by the presence or absence of AF (Figure 2), metoprolol treatment was associated with lower 30-month all-cause mortality among patients without AF (HR 0.69, 95% CI 0.48–0.99; log-rank *p* = 0.041). In contrast, no statistically significant difference in mortality was observed between treatment groups among patients with AF (HR 0.79, 95% CI 0.57–1.09; log-rank *p* = 0.155) (*p* for interaction = 0.575). In a further exploratory analysis, patients were stratified according to the percentage of target β-blocker dose prescribed at discharge (0–12.5%, >12.5–25%, >25–50%, and >50% of target dose). Across dose categories, no statistically significant difference in 30-month survival was observed (log-rank *p* = 0.260; Figure 3). A trend toward improved survival with higher dose categories was noted for the secondary endpoint; however, this did not reach statistical significance (log-rank *p* = 0.064). Given the limited number of patients and events within individual dose strata, these findings should be interpreted cautiously and considered hypothesis-generating.

### 3.3. Multivariable Cox Regression Analyses

**Figure 4:**
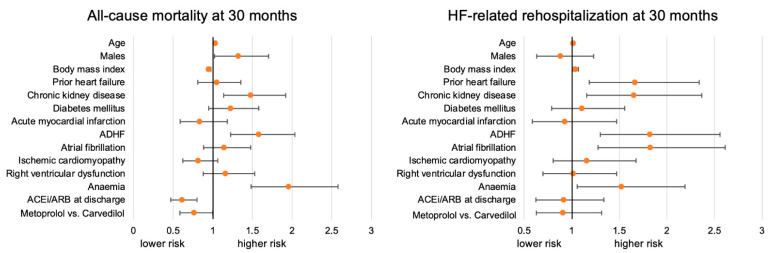
Forest plots from multivariable Cox regression analyses estimating the risk of 30-month all-cause mortality (**left panel**) and HF-related rehospitalization (**right panel**).

After multivariable adjustments, β-blocker type demonstrated a directionally favorable association for metoprolol versus carvedilol (aHR 0.76, 95% CI 0.58–1.00) (Figure 4). Furthermore, mortality risk increased with age (aHR 1.03 per year increase, 95% CI 1.021–1.046) and was higher in males (aHR 1.32, 95% CI 1.02–1.71), in patients with prior chronic kidney disease (aHR 1.48, 95% CI 1.14–1.92), with acute decompensated HF (aHR 1.58, 95% CI 1.23–2.04), and with anaemia (aHR 1.96, 95% CI 1.48–2.58). By contrast, BMI showed an inverse association with mortality (aHR 0.95 per kg/m^2^ increase, 95% CI 0.92–0.97). Prescription of an ACEi or angiotensin receptor blocker at discharge was independently associated with lower mortality (aHR 0.61, 95% CI 0.47–0.80). Other covariates, including prior HF, diabetes mellitus, acute myocardial infarction, AF, ischaemic cardiomyopathy, and right ventricular dysfunction, were not independently associated with 30-month mortality. The Schoenfeld residuals showed no statistically significant deviations from proportional hazards for any of the covariates, including age (*p* = 0.281), sex (*p* = 0.496), BMI (*p* = 0.111), prior heart failure (*p* = 0.569), chronic kidney disease (*p* = 0.461), diabetes (*p* = 0.098), myocardial infarction (*p* = 0.207), acute decompensated HF (*p* = 0.379), atrial fibrillation (*p* = 0.739), ischemic cardiomyopathy (*p* = 0.687), right ventricular dysfunction (*p* = 0.267), anemia (*p* = 0.374), use of ACE inhibitors or angiotensin receptor blockers (*p* = 0.187), and metoprolol versus carvedilol (*p* = 0.551).

By contrast, no association of the β-blocker type and the risk of HF-related rehospitalization at 30 months was demonstrated. In line, Schoenfeld residuals were not statistically significant for all variables regarding the risk of HF-related rehospitalization.

### 3.4. Prognostic Impact of Metoprolol Versus Carvedilol After Propensity Score Matching

**Figure 5:**
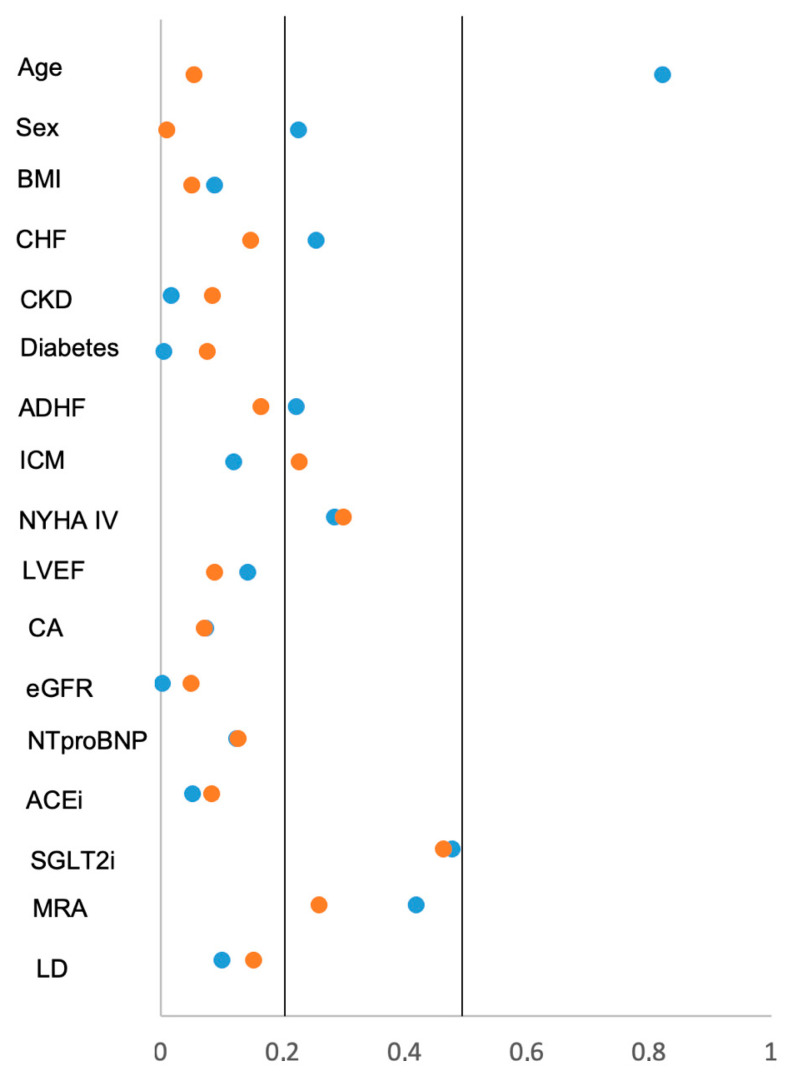
Love plot illustrating the distribution of standardized mean differences assessed by Cohen’s d for key baseline characteristics and comorbidities comparing patients with metoprolol and carvedilol. ACEi, angiotensin-converting-enzyme inhibitor; ADHF, acute decompensated heart failure; BMI, body mass index; CA, coronary angiography; CHF, congestive heart failure; eGFR, estimated glomerular filtration rate; ICM, ischemic cardiomyopathy; LD, loop diuretics; LVEF, left ventricular ejection fraction; MRA, minderalocorticoid receptor antagonist; NYHA, New York Heart Association; SGLT2i, sodium glucose linked transporter 2 inhibitor. Blue dots illustrate d before propensity score matching and orange dots thereafter.

To account for potential confounding due to imbalances in baseline characteristics, propensity score matching was performed in a 1:1 ratio. Based on predefined clinical variables, 246 patients treated with metoprolol were successfully matched with 246 patients treated with carvedilol, resulting in a propensity score-matched cohort of 492 patients (Table 1, right column). After matching, baseline characteristics were well balanced between both groups with regard to the distribution of age, sex, prior congestive HF, vital signs on admission (Table 2, right column; Figure 5). By contrast, the rates of NYHA IV, treatment with SGLT2i and aldosterone antagonists still differed significantly among both groups.

**Figure 6:**
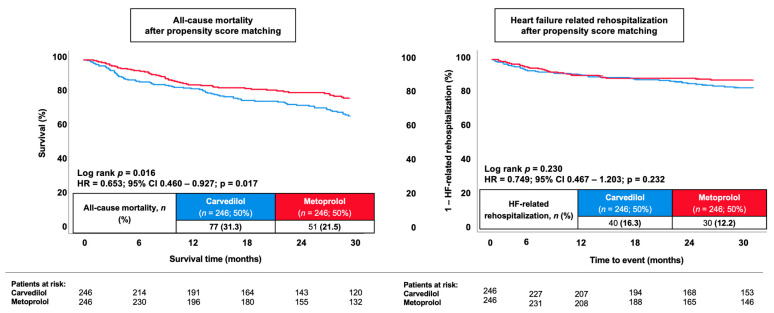
Kaplan–Meier curves comparing metoprolol and carvedilol in the propensity score-matched cohort for 30-month all-cause mortality (**left panel**) and HF-related rehospitalization (**right panel**).

In the propensity score-matched cohort, all-cause mortality at 30 months occurred in 21.5% of patients treated with metoprolol and in 31.3% of patients treated with carvedilol (HR 0.65, 95% CI 0.46–0.93; *p* = 0.017; Figure 6). Consistent results were observed in the sensitivity analysis using IPTW (metoprolol vs. carvedilol: HR = 0.748; *p* = 0.741−0.754; *p* = 0.001). In contrast, HF-related rehospitalization did not differ significantly between groups after matching (12.2% in the metoprolol group vs. 16.3% in the carvedilol group; HR 0.75, 95% CI 0.47–1.20; *p* = 0.232; Figure 6; Table 2, right column).

## 4. Discussion

In this single-center cohort of patients hospitalized with HFmrEF, patients treated with carvedilol were younger, but presented with more cardiovascular comorbidities and suffered from more advanced stages of HF. Treatment with metoprolol at index hospital discharge was associated with lower 30-month all-cause mortality compared with carvedilol after multivariable adjustment and propensity score matching. By contrast, no significant differences were observed for the risk of HF-related rehospitalization. In exploratory sub-analyses stratified by the presence or absence of AF, the association between metoprolol and lower mortality was observed among patients without AF, whereas no significant difference was found in those with AF. Further prospective and randomized studies are needed to investigate an independent effect of different β-blocker types in HFmrEF.

Whereas the prognostic impact of different HF pharmacotherapies has been predominantly investigated in HFrEF patients within RCT, each demonstrating superior outcomes in specific patient populations, which may depend on the prevalence of distinct comorbidities [11], the impact of HF pharmacotherapies in HFmrEF patients remains poorly investigated and relies predominantly on registry-based datasets. For instance, the overall benefit of β-blockers in HFmrEF remains less well established than in HFrEF, these agents are widely prescribed in routine clinical practice. Recent RCT in post-myocardial infarction patients with preserved or mildly reduced ejection fraction (LVEF ≥ 40%) have further questioned the prognostic benefit of long-term β-blocker therapy outside classical HFrEF populations, underscoring the uncertainty regarding their outcome effect in the HFmrEF range [12,13]. In this context, evaluating potential agent-specific associations may provide clinically relevant, hypothesis-generating insights. However, given the observational design and potential residual confounding, our findings should not be interpreted as evidence of superiority of one β-blocker over another but rather as exploratory data that may inform future randomized investigations.

Several established prognostic markers were independently associated with mortality, including older age, male sex, chronic kidney disease, anaemia, and acute decompensated HF at index admission. These findings are consistent with prior HF literature and support the internal validity of the model. Prescription of ACEi/ARB at discharge was independently associated with lower mortality, reinforcing the importance of guideline-directed medical therapy (GDMT) in HFmrEF as recommended by current European guidelines [4], even in patients with HFmrEF.

The present findings differ from the COMET trial, in which carvedilol reduced all-cause mortality compared with metoprolol tartrate in patients with HFrEF [9]. However, important differences must be considered. COMET enrolled predominantly patients with HFrEF, whereas our cohort consisted of HFmrEF patients, a heterogeneous phenotype with overlapping features of HFrEF and HFpEF [4]. However, the findings of the COMET trial align with the distribution of baseline characteristics and comorbidities, suggesting more advanced stages and symptoms of HF in patients treated with carvedilol, even after propensity score matching. From a pathophysiological perspective, COMET compared carvedilol with metoprolol tartrate, whereas contemporary clinical practice predominantly uses metoprolol succinate. This relates to its shorter acting than metoprolol succinate questioning a 24 h β-blockade within patients treated with metoprolol tartrate within the COMET trial [10]. To what extend the additional alpha1-blockade may improve outcomes as compared to β-blockade within HF patients remains still unclear, as doxazosin did not demonstrate improvement in hemodynamic measurements when added to metoprolol [14]. However, it must be considered that differences in phenotype, drug formulation, titration strategies, and background therapy may account for the divergent mortality signals observed between studies.

HFmrEF is increasingly recognized as an intermediate phenotype with biological and clinical overlap with both HFrEF and HFpEF [4]. Evidence from pooled analyses of PARADIGM-HF and PARAGON-HF suggests greater benefit of neurohormonal blockade within the mid-range LVEF spectrum [15,16]. Similarly, SGLT2i have demonstrated consistent reductions in cardiovascular events across LVEF > 40% in EMPEROR-Preserved and DELIVER [7,17]. These data collectively emphasize that optimization of comprehensive GDMT remains the cornerstone of HFmrEF management. In clinical practice, our findings do not establish superiority of one β-blocker over another in HFmrEF. Rather, they suggest that differential associations may exist according to rhythm status. However, given the observational design, potential residual confounding, and borderline adjusted estimates, these data should not be interpreted as supporting a definitive treatment preference.

The rhythm-dependent pattern observed in our subgroup analyses aligns with prior individual patient data meta-analyses demonstrating a mortality benefit of β-blockers in sinus rhythm but not in atrial fibrillation [18]. In AF, the loss of atrioventricular synchrony and beat-to-beat irregularity may attenuate the prognostic effect of β-blockade beyond rate control. Whereas an association of metoprolol with lower mortality was demonstrated in patients without AF, this subgroup results should be interpreted cautiously given the limited number of events.

Taken together, metoprolol was associated with lower 30-month mortality compared with carvedilol in this observational HFmrEF cohort, particularly among patients without AF, while the risk of HF-related rehospitalization were comparable. These findings should be regarded as hypothesis-generating and warrant confirmation in randomized or pragmatic comparative-effectiveness studies specifically targeting HFmrEF populations, specifically given the overall heterogenous distribution of baseline characteristics and comorbidities in patients treated with carvedilol or metoprolol within the present study.

### Study Limitations

This study has several limitations. First, due to its retrospective single-center design, the analysis is subject to both measured and unmeasured confounding despite multivariable adjustment and propensity score matching. Residual confounding by indication cannot be excluded, and causal inference is therefore not possible. Specifically, the distribution of NYHA functional class, as well as MRA and SGLT2i differed significantly in the matched cohort. Therefore, the findings should be interpreted as hypothesis-generating. Second, patients who died during the index hospitalization were excluded, as exposure was defined at hospital discharge. This may have introduced survivor bias, and the present findings therefore apply only to patients discharged alive. Third, exposure was defined based on β-blocker therapy prescribed at hospital discharge. Longitudinal information on treatment modification, including initiation, discontinuation, switching, or dose titration during follow-up, was not systematically available and therefore beyond the scope of the study. Consequently, actual pharmacological exposure over time may have differed from discharge prescriptions, potentially introducing exposure misclassification. Fourth, HF-related rehospitalizations were captured exclusively at our institution. Events occurring at external hospitals were not systematically recorded and may have led to underestimation of rehospitalization rates and potential misclassification of outcomes. Fifth, adverse effects related to HF pharmacotherapies were not systematically documented within the registry. Finally, detailed causes of death beyond the index hospitalization were not available, precluding differentiation between cardiovascular and non-cardiovascular mortality.

## 5. Conclusions

In this single-centre observational cohort of patients with HFmrEF, discharge treatment with metoprolol was associated with lower 30-month all-cause mortality compared with carvedilol, whereas HF-related rehospitalization did not differ between groups. Given the retrospective design, potential residual confounding, single-centre event capture, and borderline adjusted estimates, these findings should be interpreted with caution. The observed associations are hypothesis-generating and do not support a practice-changing recommendation. Prospective randomized or pragmatic comparative-effectiveness studies are warranted to clarify potential agent-specific differences within contemporary guideline-directed medical therapy.

## Figures and Tables

**Table 1:** Baseline characteristics.

	*Before Propensity Score Matching*					*After Propensity Score Matching*				
	Carvedilol (*n* = 283)		Metoprolol (*n* = 1033)		*d*	Carvedilol (*n* = 246)		Metoprolol (*n* = 246)		*d*
**Age**, median (IQR)	72	(60–81)	76	(65–83)	0.823	72	(60–80)	73	(61–80)	0.056
**Male sex**, *n* (%)	206	(72.8)	661	(64.0)	0.226	175	(71.1)	171	(69.9)	0.011
**Body mass index,** kg/m^2^, median (IQR)	27	(23–31)	27	(24–31)	0.089	27	(23–31)	27	(24–31)	0.052
**SBP**, mmHg, median (IQR)	145	(129–163)	140	(125–161)	0.014	145	(127–162)	139	(124–160)	0.068
**DBP**, mmHg, median (IQR)	80	(70–93)	80	(70–92)	0.028	80	(70–91)	80	(70–90)	0.011
**Heart rate**, bpm, median (IQR)	81	(68–98)	81	(69–98)	0.025	81	(68–98)	85	(70–100)	0.114
**Medical history**, *n* (%)										
Coronary artery disease	120	(42.4)	441	(42.7)	0.007	107	(43.5)	99	(40.2)	0.074
Prior myocardial infarction	73	(25.8)	267	(25.8)	0.002	64	(26.0)	64	(26.0)	0
Prior PCI	80	(28.3)	316	(30.6)	0.062	71	(28.9)	77	(31.3)	0.064
Prior CABG	25	(8.8)	112	(10.8)	0.125	21	(8.5)	27	(11.0)	0.154
Prior valvular surgery	15	(5.3)	44	(4.3)	0.127	11	(4.5)	11	(4.5)	0
Congestive heart failure	125	(44.2)	343	(33.2)	0.256	110	(44.7)	94	(38.2)	0.148
Stable heart failure	25	(8.8)	89	(8.6)	0.015	24	(9.8)	30	(12.2)	0.138
Improved heart failure	26	(9.2)	30	(2.9)	0.672	21	(8.5)	9	(3.7)	0.496
Deteriorated heart failure	40	(14.1)	106	(10.3)	0.201	35	(14.2)	26	(10.6)	0.187
Not documented	34	(12.0)	118	(11.4)	0.032	30	(12.2)	29	(11.8)	0.021
Prior ICD	6	(2.1)	27	(2.6)	0.118	6	(2.4)	7	(2.8)	0.087
Chronic kidney disease	93	(32.9)	322	(31.2)	0.018	77	(31.3)	69	(28.0)	0.086
Peripheral artery disease	25	(8.8)	117	(11.3)	0.152	22	(8.9)	34	(13.8)	0.270
Stroke	34	(12.0)	158	(15.3)	0.154	28	(11.4)	42	(17.1)	0.260
Liver cirrhosis	9	(3.2)	16	(1.5)	0.406	8	(3.3)	2	(0.8)	0.778
Malignancy	38	(13.4)	138	(13.4)	0.003	34	(13.8)	35	(14.2)	0.019
COPD	43	(15.2)	112	(10.8)	0.214	37	(15.0)	37	(15.0)	0
**Cardiovascular risk factors**, *n* (%)										
Arterial Hypertension	216	(76.3)	818	(79.2)	0.091	183	(74.4)	192	(78.0)	0.112
Diabetes mellitus	104	(36.7)	377	(36.5)	0.006	86	(35.0)	94	(38.2)	0.077
Hyperlipidemia	93	(32.9)	332	(32.1)	0.018	85	(34.6)	79	(32.1)	0.061
Smoking	120	(42.4)	390	(37.8)	0.107	102	(41.5)	104	(42.3)	0.201
Current	71	(25.1)	198	(19.2)	0.190	63	(25.6)	64	(26.0)	0.012
Former	49	(17.3)	192	(18.6)	0.048	39	(15.9)	40	(16.3)	0.017
Family history	41	(14.5)	110	(10.6)	0.187	36	(14.6)	31	(12.6)	0.095
**Comorbidities**, *n* (%)										
Unstable angina	18	(6.4)	47	(4.5)	0.195	15	(6.1)	10	(4.1)	0.235
STEMI	28	(9.9)	121	(11.7)	0.104	27	(11.0)	39	(15.9)	0.234
NSTEMI	32	(11.3)	156	(15.1)	0.096	27	(11.0)	41	(16.7)	0.267
ADHF < 12 months	84	(29.7)	227	(22.0)	0.223	70	(28.5)	56	(22.8)	0.165
Cardiogenic shock	6	(2.1)	28	(2.7)	0.139	5	(2.0)	6	(2.8)	0.190
Atrial fibrillation	119	(42.0)	485	(47.0)	0.032	97	(39.4)	109	(44.3)	0.111
Stroke	27	(9.5)	105	(10.2)	0.039	21	(8.5)	22	(8.9)	0.028
**Medication on admission**, *n* (%)										
ACE-inhibitor	100	(35.3)	392	(37.9)	0.063	81	(32.9)	97	(39.4)	0.156
ARB	70	(24.7)	228	(22.1)	0.082	60	(24.4)	49	(19.9)	0.143
Beta-blocker	161	(56.9)	687	(66.5)	0.225	138	(56.1)	165	(67.1)	0.257
MRA	54	(19.1)	76	(7.4)	0.600	49	(19.9)	22	(8.9)	0.512
ARNI	7	(2.5)	8	(0.8)	0.650	7	(2.8)	3	(1.2)	0.481
SGLT2-inhibitor	15	(5.3)	14	(1.4)	0.774	13	(5.3)	3	(1.2)	0.832
Loop diuretics	115	(40.6)	381	(36.9)	0.087	94	(38.2)	81	(32.9)	0.127
Statin	134	(47.3)	492	(47.6)	0.006	116	(47.2)	119	(48.4)	0.027
ASA	92	(32.5)	343	(33.2)	0.017	84	(34.1)	82	(33.3)	0.020
P2Y12-inhibitor	38	(13.4)	98	(9.5)	0.216	34	(13.8)	34	(13.8)	0
DOAC	69	(24.4)	267	(25.8)	0.043	58	(23.6)	58	(23.6)	0
VKA	32	(11.3)	90	(8.7)	0.160	26	(10.6)	17	(6.9)	0.256

ACE, angiotensin-converting-enzyme; ADHF, Acute Decompensated heart failure; ARB, angiotensin receptor blocker; ARNI, angiotensin receptor neprilysin inhibitor; ASA, acetylsalicylic acid; CABG, coronary artery bypass grafting; COPD, chronic obstructive pulmonary disease; DBP, diastolic blood pressure; DOAC, directly acting oral anticoagulant; IQR, interquartile range; MRA, mineralocorticoid receptor antagonist; (N)STEMI, (non-)ST-segment elevation myocardial infarction; SBP, systolic blood pressure; SGLT2, sodium glucose linked transporter 2; ICD, implantable cardioverter defibrillator; VKA, Vitamin K antagonist.

**Table 2:** Heart failure related and procedural data.

	*Before Propensity Score Matching*			*After Propensity Score Matching*		
	Carvedilol (*n* = 283)	Metoprolol (*n* = 1033)	*d*	Carvedilol (*n* = 246)	Metoprolol (*n* = 246)	*d*
**Heart failure etiology**, *n* (%)						
Ischemic cardiomyopathy	169 (59.7)	670 (64.9)	0.121	149 (60.6)	172 (69.9)	0.228
Non-ischemic cardiomyopathy	39 (13.8)	47 (4.5)	0.667	34 (13.8)	13 (5.3)	0.582
Hypertensive cardiomyopathy	14 (4.9)	71 (6.9)	0.193	11 (4.5)	13 (5.3)	0.097
Congenital heart disease	1 (0.4)	1 (0.1)	0	1 (0.4)	0 (0.0)	0.608
Valvular heart disease	17 (6.0)	29 (2.8)	0.438	15 (6.1)	6 (2.4)	0.526
Tachycardia-associated	12 (4.2)	86 (8.3)	0.396	3 (1.2)	9 (3.7)	0.617
Tachymyopathy	6 (2.1)	28 (2.7)	0.139	5 (2.0)	9 (3.7)	0.333
Pacemaker-induced cardiomyopathy	1 (0.4)	7 (0.7)	0.361	1 (0.4)	0 (0.0)	0.608
Unknown	30 (10.6)	122 (11.8)	0.067	27 (11.0)	24 (9.8)	0.072
**NYHA functional class**, *n* (%)						
I/II	187 (66.1)	748 (72.4)	0.164	167 (67.9)	177 (72.0)	0.107
III	61 (21.6)	205 (19.8)	0.057	49 (19.9)	50 (20.3)	0.014
IV	35 (12.4)	80 (7.7)	0.286	31 (12.6)	19 (7.7)	0.300
**Echocardiographic data**						
LVEF, %, median (IQR)	45 (43–47)	45 (45–47)	0.143	45 (43–47)	45 (45–47)	0.089
IVSd, mm, median (IQR)	12 (10–13)	12 (11–13)	0.061	12 (10–13)	12 (11–13)	0.114
LVEDD, mm, median (IQR)	50 (45–54)	48 (44–53)	0.209	50 (45–54)	48 (44–53)	0.172
TAPSE, mm, median (IQR)	20 (18–23)	20 (17–23)	0.023	20 (18–23)	20 (17–23)	0.055
Diastolic dysfunction, n (%)	196 (69.3)	744 (72.0)	0.074	169 (68.7)	163 (66.3)	0.061
Moderate/severe aortic stenosis, *n* (%)	27 (9.5)	86 (8.3)	0.083	24 (9.8)	21 (8.5)	0.081
Moderate/severe aortic regurgitation, *n* (%)	15 (5.3)	34 (3.3)	0.268	12 (4.9)	12 (4.9)	0
Moderate/severe mitral regurgitation, *n* (%)	40 (14.1)	114 (11.0)	0.156	38 (15.4)	31 (12.6)	0.131
Moderate/severe tricuspid regurgitation, *n* (%)	47 (16.6)	152 (14.7)	0.079	40 (16.3)	31 (12.6)	0.164
**Coronary angiography**, *n* (%)	145 (51.2)	494 (47.8)	0.075	127 (51.6)	135 (54.9)	0.072
No evidence of coronary artery disease	29 (20.0)	76 (15.4)	0.176	26 (20.5)	16 (11.9)	0.358
1-vessel disease	36 (24.8)	95 (19.2)	0.180	33 (26.0)	29 (21.5)	0.138
2-vessel disease	24 (16.6)	118 (23.9)	0.253	21 (16.5)	36 (26.7)	0.335
3-vessel disease	56 (38.6)	205 (41.5)	0.067	47 (37.0)	54 (40.0)	0.070
CABG	11 (7.6)	41 (8.3)	0.054	5 (3.9)	9 (6.7)	0.306
Chronic total occlusion	19 (13.1)	67 (13.6)	0.022	14 (11.0)	26 (19.3)	0.361
PCI, n (%)	73 (50.3)	297 (60.1)	0.219	64 (50.4)	84 (62.2)	0.267
**Laboratory values**, median (IQR)						
Potassium, mmol/L	3.9 (3.6–4.2)	3.9 (3.6–4.2)	0	3.9 (3.6–4.2)	3.9 (3.6–4.2)	0
Sodium, mmol/L	139 (137–141)	139 (137–141)	0.111	139 (137–141)	139 (137–141)	0.117
Creatinine, mg/dL	1.09 (0.91–1.52)	1.07 (0.86–1.44)	0.447	1.07 (0.80–1.34)	1.08 (0.77–1.39)	0.179
eGFR, mL/min/1.73 m^2^	64 (45–85)	65 (45–85)	0.003	68 (46–90)	65 (45–86)	0.050
Hemoglobin, g/dL	12.3 (10.4–14.0)	12.5 (10.6–14.1)	0.043	12.6 (10.8–14.5)	12.3 (10.5–14.1)	0.083
WBC count, ×10^9^/L	7.90 (6.19–9.90)	8.31 (6.63–10.21)	0.102	8.6 (6.7–10.5)	8.0 (6.1–9.9)	0.157
Platelet count, ×10^9^/L	224 (175–276)	225 (180–290)	0.130	234 (173–296)	220 (165–275)	0.183
HbA1c, %	5.9 (5.5–6.6)	5.9 (5.5–6.8)	0	6.0 (5.5–6.5)	5.9 (5.4–6.4)	0.143
LDL-cholesterol, mg/dL	99 (76–129)	97 (74–124)	0.111	101 (73–130)	98 (72–125)	0.003
HDL-cholesterol, mg/dL	40 (35–51)	42 (34–51)	0.080	40 (33–50)	41 (33–50)	0.029
C-reactive protein, mg/L	13 (4–45)	13 (3–41)	0.058	12 (3–35)	13 (3–33)	0.048
NT-pro BNP, pg/mL	2370 (1209–5392)	2634 (1084–6939)	0.125	2700 (100–6200)	2264 (100–4424)	0.128
Cardiac troponin I, µg/L	0.03 (0.02–0.24)	0.04 (0.02–0.30)	0.001	0.05 (0–0)	0.03 (0–0)	0.079
**Medication at discharge**, *n* (%)						
ACE-inhibitor	165 (58.3)	578 (56.0)	0.053	141 (57.3)	155 (63.0)	0.084
ARB	67 (23.7)	242 (23.4)	0.008	60 (24.4)	55 (22.4)	0.063
MRA	71 (25.1)	140 (13.6)	0.419	61 (24.8)	42 (17.1)	0.260
ARNI	10 (3.5)	11 (1.1)	0.675	10 (4.1)	3 (1.2)	0.680
SGLT2-inhibitor	23 (8.1)	37 (3.6)	0.478	22 (8.9)	10 (4.1)	0.464
Loop diuretics	154 (54.4)	515 (49.9)	0.101	129 (52.4)	112 (45.5)	0.153
Thiazide diuretics	48 (17.0)	188 (18.2)	0.047	45 (18.3)	39 (15.9)	0.101
Statin	199 (70.3)	776 (75.1)	0.134	173 (70.3)	193 (78.5)	0.237
Digitalis	12 (4.2)	61 (5.9)	0.192	9 (3.7)	12 (4.9)	0.166
Amiodarone	10 (3.5)	33 (3.2)	0.058	8 (3.3)	7 (2.8)	0.076
ASA	133 (47.0)	547 (53.0)	0.132	121 (49.2)	135 (54.9)	0.126
P2Y12-inhibitor	104 (36.7)	389 (37.7)	0.021	93 (37.8)	107 (43.5)	0.130
DOAC	97 (34.3)	390 (37.8)	0.083	82 (33.3)	90 (36.6)	0.079
VKA	30 (10.6)	72 (7.0)	0.253	25 (10.2)	14 (5.7)	0.368

ACE, angiotensin-converting enzyme; ARB, angiotensin receptor blocker; ARNI, angiotensin receptor neprilysin inhibitor; ASA, acetylsalicylic acid; CABG, coronary artery bypass grafting; DOAC, directly acting oral anticoagulant; eGFR, estimated glomerular filtration rate; HbA1c, glycated haemoglobin; HDL, high-density lipoprotein; IQR, interquartile range; IVSd, Interventricular septal end diastole; LDL, low-density lipoprotein; LVEDD, Left ventricular end-diastolic diameter; LVEF, left ventricular ejection fraction; MRA, mineralocorticoid receptor antagonist; NT-pro BNP, aminoterminal pro-B-type natriuretic peptide; NYHA, New York Heart Association; PCI, percutaneous coronary intervention; TAPSE, tricuspid annular plane systolic excursion; VKA, Vitamin K antagonist; WBC, white blood cells.

## Data Availability

The datasets used and/or analyzed during the current study are available from the corresponding author upon reasonable request.
